# Determining the association of hyperoxia while on extracorporeal life support with mortality in neonates following Norwood operation

**DOI:** 10.1051/ject/2024020

**Published:** 2024-12-20

**Authors:** Asaad G. Beshish, Alaa Aljiffry, Yijin Xiang, Sean Evans, Amy Scheel, Ashley Harriott, Shayli Patel, Alan Amedi, Amanda Harding, Joel Davis, Subhadra Shashidharan, David M. Kwiatkowski

**Affiliations:** 1 Department of Pediatrics, Division of Cardiology, Emory University School of Medicine, Children’s Healthcare of Atlanta Atlanta GA USA; 2 Biostatistician and Data Analyst, Department of Pediatrics, Emory University School of Medicine Atlanta GA USA; 3 Emory University School of Medicine Atlanta GA USA; 4 Senior Pediatric Cardiac Sonographer, Children’s Healthcare of Atlanta Atlanta GA USA; 5 Advanced Technology Coordinator, ECMO and Advanced Technologies, Children’s Healthcare of Atlanta Atlanta GA USA; 6 Department of Surgery, Division of Cardiothoracic Surgery, Emory University School of Medicine, Children’s Healthcare of Atlanta Atlanta GA USA; 7 Department of Pediatrics, Division of Cardiology, Stanford University School of Medicine, Lucile Packard Children’s Hospital Stanford CA USA

**Keywords:** Univentricular Physiology, Norwood Operation, Extracorporeal Life Support (ECLS), Hyperoxia, Functional Status Scale (FSS), New Morbidity, Unfavorable Outcomes

## Abstract

*Background*: Patients requiring extracorporeal life support (ECLS) support post-Norwood operation constitute an extremely high-risk group. *Materials and methods*: We retrospectively aimed to evaluate the relationship of hyperoxia with mortality and other clinical outcomes in patients who required ECLS following Norwood operation between January/2010 and December/2020 in a large volume center. *Results*: During the study period 65 patients required ECLS post-Norwood. Using receiver operating characteristic (ROC) curve analysis, mean PaO_2_ of 182 mmHg in the first 48-hour on ECLS was determined to have the optimal discriminatory ability for mortality (sensitivity 68%, specificity 70%). Of the 65 patients, 52% had PaO_2_ > 182 mmHg and were designated as hyperoxia group. Patients in the hyperoxia-group had longer cardiopulmonary bypass time (187 vs. 165 min, *p* = 0.023), shorter duration from CICU arrival to ECLS-cannulation (13.28 vs. 132.58 h, *p* = 0.003), higher serum lactate within 2-hours from ECLS-canulation (14.55 vs. 5.80, *p* = 0.01), higher ECLS flows in the first 4-hours (152.68 vs. 124.14, *p* = 0.006), and higher mortality (77% vs. 39%, *p* = 0.005). In the unadjusted-analysis, using a derived cut-point, patients in the hyperoxia-group had 5.15 higher odds of mortality (*p* = 0.003). However, this association was insignificant when adjusting for confounding variables (*p* = 0.104). Using a functional status scale, new morbidity (38% vs. 21%), and unfavorable outcomes (13% vs. 5%) were higher in the hyperoxia group. Despite being higher in the hyperoxia group, this did not reach statistical significance. *Conclusion*: Neonates with hyperoxia (PaO_2_ > 182 Torr) during the first 48-hour of ECLS post-Norwood operation had 5 times higher odds of mortality in the unadjusted analysis, however, this was insignificant when adjusting for confounding variables. Patients in the hyperoxia group had shorter duration from CICU arrival to ECLS-cannulation, higher serum lactate prior to ECLS-canulation, and higher ECLS flows in the first 4-hours, (*p* < 0.05). Multicenter evaluation of this modifiable risk factor is imperative to improve the care of this high-risk cohort.

## Introduction

The use of extracorporeal life support (ECLS) following cardiac surgery is utilized in 3–5% of all neonatal and pediatric cardiac surgeries with a 43–52% survival [1, 2]. Neonates with univentricular physiology who undergo a Norwood operation represent an especially high-risk population [3–6]. The tenuous nature of their postoperative physiology requires significant cardiopulmonary support and at times ECLS to maintain adequate hemodynamics and oxygen delivery. Previous reports indicate that 8–22% of neonates will require ECLS post-Norwood operation [4, 5, 7–9], with an overall survival rate of 30–60% [4, 5, 7–9].

When ECLS is used postoperatively, patients are supported with venoarterial-ECLS. These ECLS circuits utilize an efficient oxygenator resulting in high partial pressure of oxygen (PaO_2_) that can exceed 400 mmHg. Exposure to supranormal levels of oxygen is termed hyperoxia. Hyperoxia has been well studied in different clinical situations, including after resuscitation from cardiac arrest, perinatal asphyxia, myocardial infarctions, traumatic brain injury, and following cardiopulmonary bypass (CPB). Studies in both adults and children have demonstrated an association with increased morbidity and mortality [1, 10–18]. Furthermore, minimizing PaO_2_ while on CPB in cyanotic patients with complete mixing congenital heart lesions has been shown to result in less end-organ damage, inflammation, and oxidative stress when compared to those exposed to higher oxygen concentrations [1, 19, 20]. Although the negative effect of hyperoxia and its association with adverse outcomes is known, adequate oxygen delivery is necessary and the level at which PaO_2_ may become deleterious may differ depending on the clinical situation, duration of exposure, patient’s age, underlying pathophysiology, and disease process [1, 10, 14, 21–23].

Given the lack of a clear definition of hyperoxia, we aimed to evaluate a high-risk homogenous patient population, specifically neonates with univentricular physiology who underwent Norwood operation and required ECLS in the postoperative period. Our primary aim was to determine if hyperoxia while on ECLS is associated with mortality using a derived cut-point within our cohort. Our secondary aim was to determine if hyperoxia during ECLS was associated with morbidity including Functional Status Scale (FSS), acute kidney injury (AKI), and prolonged postoperative length of stay (PPLOS).

## Materials and methods

This is a single-center retrospective cohort study including all neonates with univentricular physiology who underwent a Norwood operation and required ECLS in the postoperative period between January 1st, 2010, and December 31st, 2020, at Children’s Healthcare of Atlanta (CHOA), a large quaternary children’s hospital. An internal surgical and ECLS database/registry were queried, and eligible patients were identified. Procedures were followed in accordance with the ethical standards of the CHOA Institutional Review Board (IRB# 00001119, approval 07/19/2021), and the Helsinki Declaration of 1975. Informed consent was waived.

### Data and definitions

All consecutive patients who underwent Norwood operation and required ECLS in the index postoperative ICU hospitalization were included. Patients who failed to separate from CPB and initiated ECLS in the operating room were excluded (*n* = 6). Also, patients who underwent a hybrid procedure prior to undergoing a Norwood’ operation were excluded from our study analysis (*n* = 3). These 2 exclusion criteria were chosen to make the patient population in the study as homogenous as possible. None of our patients underwent a concomitant AVV repair at the time of Norwood’s operation. Demographic features and clinical characteristics, including chromosomal abnormalities, genetic syndrome, primary cardiac diagnosis, type of systemic ventricle, source of pulmonary blood flow [modified Blalock-Taussig-Thomas (m-BTT) shunt vs. Sano shunt/right ventricle to pulmonary artery (RV-PA) conduit], preoperative respiratory support, creatinine, preoperative and intraoperative echocardiogram [assessing atrioventricular valve regurgitation (AVVR), systemic ventricular function, and ascending aorta diameter], preoperative and postoperative vasoactive inotropic scores (VIS) at dedicated postoperative times points; operative variables, including CPB, aortic cross-clamp (XC), and circulatory arrest times; and ECLS variables (cannulation site, ECLS duration, and ECLS complications) were collected. All arterial blood gases (ABGs) were obtained from the patient’s right radial arterial line (institutional standard of care includes placement of a right radial arterial line to monitor hemodynamics and arterial blood gases) during and after the Norwood operation. ABG data was obtained prior to ECLS initiation, and during the first 48 h while on ECLS.

### Outcomes

The primary outcome was all-cause ECLS mortality. The secondary outcomes included FSS (preoperative compared to discharge), AKI (Stage II or III, as defined by the KDIGO scoring criteria) [24], and PPLOS, defined a-priori as the fourth quartile of the postoperative length of stay (≥55 days). Length of stay may be biased by mortality, because those who die may have a shorter length of stay, which would erroneously appear as a good outcome. Two methods were used to control for this bias. In standard logistic regression, the length of stay for mortality was defined as prolonged, regardless of duration. In a second analysis, a composite rank-based outcome was created for days alive ICU-free (AIF). In this method, the AIF composite endpoint equals the number of ICU-free days over the first 28 postoperative days. Those with mortality were assigned a score of −1 [25].

### Functional Status Scale (FSS)

The FSS consists of six main domains: mental status, sensory, communications, motor function, feeding, and respiratory. Functional status for each domain was categorized from normal (1) to very severe dysfunction (5), with total FSS scores ranging from 6 to 30 [26]. Functional status scoring for this study involved retrospectively scoring baseline status (i.e., on admission) and again at hospital discharge utilizing appropriate documentation. FSS score determination was blinded from hyperoxia status. Newborns who had never achieved a stable baseline function were assigned a score of 6. This was operationalized by assigning a baseline FSS score of 6 to all admissions for infants 0–2 days old and to transfers from another facility for infants 3–6 days old as previously reported [27–30]. New morbidity was defined as an increase in the total score of ≥3 points, and unfavorable functional outcome was defined as an increase of ≥5 points [31].

### Clinical management

In our center, in the period 2010–2020, there were multiple different iterations in the layout of the ECLS circuit, and we used different manufacturers for different circuit components/parts. All circuits were blood-primed before the start of ECLS with packed red blood cells, 25% albumin, sodium bicarbonate, calcium gluconate, and heparin. It is common practice for ABGs to be obtained at the discretion of the clinical team, commonly approximately 30 min after initial ECLS cannulation, and then hourly for the first several hours. Subsequently, blood gases are obtained every 3–6 h and shortly after an adjustment in ECLS support. Target gas exchange parameters are not dictated by protocol at our center. Goal PaO_2_ ranges have no established normal and the variation we describe is derived from measurements occurring during clinical care. We target goal arterial oxygen saturations >80%, and to ensure adequacy of cardiac output, we target pre-membrane saturations >50% using Spectrum Medical Perfusion Monitor M3 and M4, Fort Mill, SC, USA. Goal PaCO_2_ was typically 35–45 mmHg, and goal pH was typically 7.35–7.45. While on ECLS, our center’s approach was not to mechanically limit or clamp any source of pulmonary blood flow (i.e., m-BTT or Sano shunt).

### Statistical analysis

Variables were described using medians with interquartile ranges (IQR) or counts with percentages. Patient characteristics were compared between hyperoxia and non-hyperoxia groups. Comparisons were made using chi‐square tests or Fisher’s exact tests for categorical variables and Wilcoxon rank-sum tests for continuous variables. Average PaO_2_ while on CPB was calculated for individual patients. The correlation coefficient with a 95% confidence interval (95% CI) was calculated. ROC curve and area under the curve (AUC) analyses were performed to identify the optimal cut-off values of mean PaO_2_ for predicting mortality within 30 days after the operation. All variables were entered into binary logistic regression analyses with pre-identified hyperoxia status as the outcome. The associations between hyperoxia status and adverse outcomes (i.e., Stage II or III AKI, PPLOS, and mortality) were assessed using binary logistics and multivariable logistics regression controlling for those explanatory variables with a *p*-value < 0.05. Odds ratios (OR) and adjusted odds ratios (aOR) with 95% CI were presented. The association between average PaO_2_ on ECLS and duration of ECLS was further assessed using Pearson correlation. Overall FSS and subdomain FSS were reported as mean and standard deviation (SD) and paired Student’s *t*-test was used to compare scores at admission and at discharge. All *p*-values < .05 were considered significant (two-tailed). All analyses were performed using SAS version 9.4 (SAS Institute, Cary, NC) and R statistical software (version 4.0.2; R Core Team, 2020).

## Results

### Patient demographics and characteristics for overall cohort

**Figure 1:**
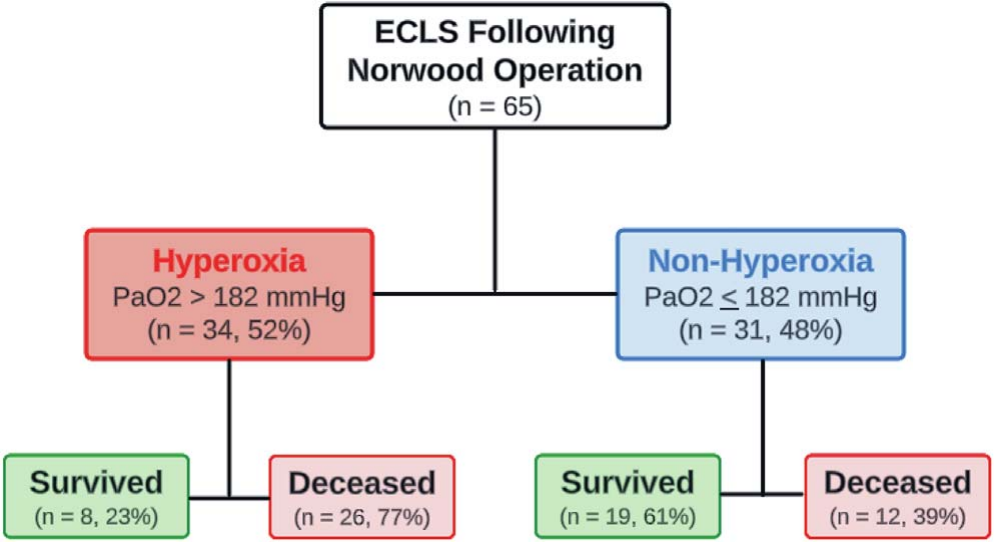
Flow chart of neonates requiring extracorporeal life support post-Norwood operation stratified based on PaO_2_ levels in the first 48-hours while on ECLS.

During the period, there were 269 neonates with univentricular physiology who underwent Norwood operation. Of these, 65/269 (24%) required ECLS support (Figure 1). The median age at the time of surgery was 6 (IQR 4, 7) days, and the median weight was 3.2 (IQR 2.8, 3.5) kg. The most frequent cardiac diagnosis was hypoplastic left heart syndrome in 50/65 (77%) of cases, and mitral atresia with aortic atresia was the most common variant in 26/50 (51%). Patient demographics and clinical characteristics are presented in Table 1. Of note, none of our patients underwent a concomitant AVV repair at the time of Norwood’s operation.

**Table 1:** Patient demographics and clinical characteristic for neonates requiring extracorporeal life support post-Norwood operation.

Variables	*n* (%), median (IQR)
Age at day of surgery (days)	6.0 (4.0, 7.0)
Weight at day of surgery (kg)	3.2 (2.8, 3.5)
Sex	
Male	40 (62%)
Female	25 (38%)
Race	
Caucasian	31 (48%)
African American	29 (45%)
Asian	0 (0%)
Hispanic	3 (5%)
Other	2 (3%)
Gestational Age (weeks)	38.0 (37.0, 39.0)
Genetic Syndrome and Chromosomal Abnormality	14 (21%)
Primary Cardiac Diagnosis	
HLHS	50 (77%)
Aortic Atresia & critical aortic Stenosis	3 (5%)
DILV	4 (6%)
DORV	1 (2%)
Single Ventricle Other	3 (5%)
Single Ventricle, unbalanced AV canal	0 (0%)
Tricuspid Atresia	4 (6%)
Single Ventricle, heterotaxia	0 (0%)
HLHS Variant	
MA/AA	26 (51%)
MA/AS	3 (6%)
MS/AA	14 (28%)
MS/AS	8 (16%)
Pre-Norwood Respiratory Support	
RA	18 (28%)
NC	14 (22%)
HFNC	10 (15%)
NIPPV	0 (0%)
Intubated	23 (35%)
Pre-Norwood Prostaglandin Dose (mcg/kg/min)	0.02 (0.01, 0.02)
Preoperative VIS-score	0.0 (0.0, 5.0)
Pre-Norwood Transthoracic Echocardiogram	
*Atrioventricular Valve Regurgitation (AVVR)*	
No – Trivial – Mild AVVR	53 (83%)
Moderate – Severe	11 (17%)
*Systemic Ventricular Function*	
Normal	35 (55%)
Mild Dysfunction	6 (9%)
Moderate – Severe Dysfunction	23 (36%)
Pre-Norwood Ascending Aorta Diameter (mm)	
Diameter (mm)	2.5 (1.9, 4.5)
Median *z*-score	−4.0 (−4.5, −2.7)
Pre-Norwood Ascending Aorta Groups Based on Diameter	
≤1.5 mm	3 (5%)
1.6–1.9 mm	16 (25%)
2.0–3.9 mm	25 (39%)
≥4.0 mm	21 (31%)
Source of Pulmonary Blood Flow	
m-BTT shunt	28 (43%)
Sano Shunt	37 (57%)
Type of systemic ventricle	
RV	52 (80.0%)
LV	12 (18%)
Undetermined	1 (2%)
Intraoperative Transesophageal Echocardiogram	
Atrioventricular Valve Regurgitation (AVVR)	
No – Trivial – Mild AVVR	40 (74%)
Moderate – Severe	14 (26%)
*Systemic Ventricular Function*	
Normal	35 (65%)
Mild Dysfunction	11 (20%)
Moderate – Severe Dysfunction	8 (15%)
Cardiopulmonary Bypass Time (min)	179.0 (157.0, 219.0)
Cross Clamp Time (min)	69.5 (57.0, 83.5)
Circulatory Arrest Time (min)	6.0 (2.0, 25.0)
Post-Norwood iNO on Arrival to CICU	17 (26%)
Post-Norwood VIS Score	
First 24 h	22.0 (15.0, 30.0)
Hours 24–48	27.0 (18.0, 38.0)
Delayed Sternal Closure	54 (83%)
Time from CICU Arrival to ECLS Cannulation (h)	94.9 (7.4, 288.4)
ECLS Cannulation Site	
Peripheral	21 (33%)
Central	43 (67%)
ECLS indication	
E-CPR	27 (41%)
Cardiac	38 (59%)
Arterial blood gas within 2 h from ECLS initiation	
Lowest pH	7.3 (7.2, 7.4)
Highest serum lactic acid	12.7 (4.6, 18.7)
Initial ECLS flow (first 4 h) (mL/kg/min)	139.5 (107.4, 163.6)
Worst arterial blood gas within 24 h from ECLS initiation	
Lowest pH	7.5 (7.4, 7.5)
Highest serum lactic acid	1.9 (1.2, 8.4)
Post-Norwood cath intervention	46 (71%)
Median PaO_2_ (mmHg)	197.1 [95.8, 289.1]
PaO_2_ range (mmHg)	
Number of PaO_2_ samples per patient	22.0 [19.0, 26.0]
Duration of mechanical ventilation (h)	414.0 (246.5, 609.1)
Stage II or III AKI based on KDIGO score	35 (54%)
Length of Stay (LOS) (days)	
CICU LOS	22.0 (13.0, 41.0)
Postoperative LOS	30.0 (16.0, 55.0)
Hospital LOS	39.0 (23.0, 64.0)
Operative mortality	38 (59%)

Results depicted in *n* (%), median (interquartile range).

Abbreviations: HLHS: Hypoplastic Left Heart Syndrome; DILV: Double Inlet Left Ventricle; DORV: Double Outlet Right Ventricle; AV Canal: Atrioventricular Canal; MA: Mitral Atresia; MS: Mitral Stenosis; AA, Aortic Atresia; AS: Aortic Stenosis; RA: Room Air; NC: Nasal Canula; HFNC: High Flow Nasal Canula; NIPPV: Non-Invasive Positive Pressure Ventilation; VIS: Vasoactive Inotropic Score; m-BTT shunt: modified Blalock-Tausig-Thomas shunt; iNO: Inhaled Nitric Oxide; CICU: Cardiac Intensive Care Unit

### Cut-point analysis

**Figure 2:**
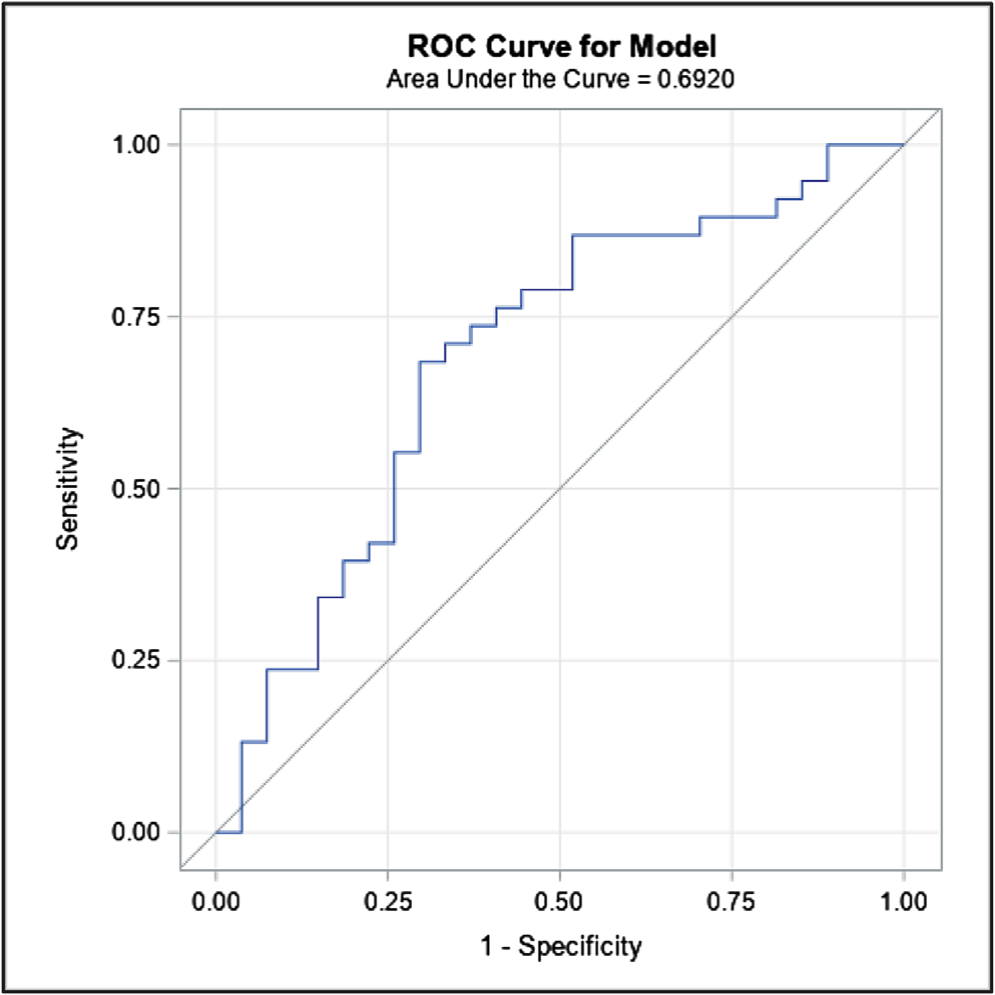
Receiver operating characteristic curve (ROC) identifying the optimal discriminatory cut-point for operative mortality was PaO_2_ = 182 mmHg (sensitivity 68%, and specificity 70%).

Using ROC analysis, PaO_2_ > 182 mmHg had the optimal discriminatory ability for operative mortality (sensitivity of 68%, and specificity of 70%) and was therefore used to define hyperoxia in our exploratory analysis (Figure 2). The AUC for average PaO_2_ during CPB and subsequent mortality was 0.69, (95% CI: 0.58–0.81; *p* = 0.001). When using the PaO_2_ > 182 mmHg threshold 34/65 were in the hyperoxia group. On univariable analyses, this designation of hyperoxia was associated with more Sano shunts/RV-PA conduits (82% vs. 29%, *p* < 0.001), had longer median CPB times (187 vs. 165 min, *p* = 0.023), had higher median VIS-scores in the first 24-hours and hours 24–48, [(25 vs. 20, *p* = 0.027), and (30 vs. 23, *p* = 0.017) respectively], higher rates of central ECLS-cannulation (85% vs. 47%, *p* = 0.003), shorter median duration from CICU arrival to ECLS-cannulation (13.3 vs. 232.6 h, *p* = 0.003), higher serum lactate within 2-hours from ECLS-canulation (14.65 vs. 5.8, *p* = 0.01), and had higher flows in the first 4-hours of ECLS (152.7 vs. 124.1, *p* < 0.05). Although the CICU, postoperative, and overall hospital length of stay was shorter in the hyperoxia group, the mortality rate was significantly higher (77% vs. 39%, *p* = 0.005) Table 2. The causes of comorbidity and death are listed in Table 3. Of the 65 patients who required post-Norwood ECLS, only 6 patients (9.2%) required reintervention [5 aortic arch augmentation/revision of DKS (Damus Kaye Stansel), and 1 patient required shunt revision].

**Table 2:** Patient demographics and clinical characteristics for neonates requiring extracorporeal life support post-Norwood operation stratified by PaO_2_ levels into hyperoxia group (PaO_2_ > 182 mmHg) and non-hyperoxia Group (PaO_2_ ≤ 182 mmHg).

Variables	Non-hyperoxia group	Hyperoxia group	*p*-value
	Average PaO_2_ £ 182	Average PaO_2_ > 182	
	(*n* = 31)	(*n* = 34)	
Age (days)	6.0 [4.0, 7.5]	6.0 (4.3, 7.0)	0.984
Weight (Kg)	3.1 [2.6, 3.5]	3.3 (2.8, 3.6)	0.641
Sex			
Male	16 (52%)	24 (71%)	0.188
Female	15 (48%)	10 (29%)	
Race			0.967
Caucasian	15 (48%)	16 (47%)	
African American	14 (45%)	15 (44%)	
Hispanic	1 (3%)	2 (6%)	
Other	1 (3%)	1 (3%)	
Gestational Age (weeks)	38.0 [37.0, 39.0]	38.5 (37.0, 39.0)	0.323
Chromosomal Abnormality and Genetic Syndrome	9 (29%)	5 (15%)	1
Primary Cardiac Diagnosis			0.226
AA & critical AS	2 (7%)	1 (3%)	
DILV	3 (10%)	1 (3%)	
DORV	1 (3%)	0 (0%)	
HLHS	22 (71%)	28 (82%)	
Single Ventricle Other	0 (0%)	3 (9%)	
Tricuspid Atresia	3 (10%)	1 (3%)	
HLHS Variant			0.2
MA/AA	11 (50%)	15 (52%)	
MA/AS	1 (5%)	2 (7%)	
MS/AA	4 (18%)	10 (35%)	
MS/AS	6 (27%)	2 (7%)	
Pre-Norwood Respiratory Support			0.327
RA	8 (26%)	10 (29%)	
NC	4 (13%)	10 (29%)	
HFNC	6 (19%)	4 (12%)	
Intubated	13 (42%)	10 (29%)	
Pre-Norwood PGE1 Dose (mcg/kg/min)	0.02 [0.01, 0.02]	0.02 (0.01, 0.03)	0.39
Preoperative VIS score	0.0 [0.0, 4.3]	1.5 (0.0, 7.8)	0.155
Pre-Norwood Transthoracic Echocardiogram			
Atrioventricular Valve Regurgitation (AVVR)			0.226
Moderate – Severe	28 (90%)	25 (76%)	
No – Trivial – Mild AVVR	3 (10%)	8 (24%)	
Systemic Ventricular Function			0.713
Normal	17 (55%)	18 (55%)	
Mild Dysfunction	2 (7%)	4 (12%)	
Moderate – Severe Dysfunction	12 (39%)	11 (33%)	
Pre-Norwood Ascending Aorta Diameter (mm)			
Diameter (mm)	3.30 [1.90, 4.80]	2.30 (1.90, 3.70)	0.148
Median *z*-score	−3.10 [− 4.44, −2.55]	−4.18 (−4.57, −3.30)	0.16
Pre-Norwood ascending aorta groups based on diameter			0.346
≤1.5 mm	1 (3%)	2 (6%)	
1.6–1.9 mm	8 (26%)	8 (24%)	
2.0–3.9 mm	9 (29%)	16 (47%)	
≥4.0 mm	13 (42%)	8 (24%)	
Source of pulmonary blood flow			**<0.001**
m-BTT shunt	22 (71%)	6 (18%)	
Sano Shunt	9 (29%)	28 (82%)	
Type of systemic ventricle			0.235
LV	8 (26%)	4 (12%)	
RV	23 (74%)	29 (85%)	
Undetermined	0 (0%)	1 (3%)	
Intraoperative transesophageal echocardiogram			
Atrioventricular Valve Regurgitation (AVVR)			0.881
Moderate – severe	20 (77%)	20 (71%)	
No – trivial – mild AVVR	6 (23%)	8 (29%)	
Systemic ventricular function			0.359
Normal	18 (69%)	17 (61%)	
Mild dysfunction	6 (23%)	5 (18%)	
Moderate – severe dysfunction	2 (8%)	6 (21%)	
Cardiopulmonary bypass time (min)	165.0 [152.0, 185.0]	187.0 (171.5, 228.3)	**0.023**
Cross clamp time (min)	70.0 [58.5, 83.0]	68.0 (57.0, 85.0)	0.952
Circulatory arrest time (min)	7.0 [2.0, 34.5]	4.0 (2.0, 23.5)	0.73
Post-Norwood iNO on arrival to CICU	8 (26%)	9 (27%)	1
Post-Norwood VIS-score			
First 24 h	20.0 [12.5, 27.0]	25.0 (20.0, 30.0)	**0.027**
Hours 24–48	23.0 [15.0, 32.5]	30.0 (21.3, 41.5)	**0.017**
Delayed sternal closure	23 (74%)	31 (91%)	0.136
Post-Norwood cath intervention	23 (74%)	23 (68%)	0.759
Median PaO_2_ (mmHg)	91.6 [67.7, 140.8]	289.0 [264.9, 337.2]	**<0.0001**
PaO_2_ range (mmHg)	62.4 – 175.8	182.4 – 412.3	**<0.0001**
Number of PaO_2_ samples per patient	22.0 [18.0, 27.0]	22.5 [19.0, 26.0]	**0.385**
Cannulation site			**0.003**
Peripheral	16 (53%)	5 (15%)	
Central	14 (47%)	29 (85%)	
ECLS indication			0.488
E-CPR	11 (36%)	16 (47%)	
Cardiac	20 (65%)	18 (53%)	
Time from CICU arrival to ECLS cannulation (h)	132.6 [42.9, 305.7]	13.3 [4.0, 74.7]	**0.003**
Arterial blood gas within 2 h from ECLS initiation			
Lowest pH	7.3 [7.2, 7.3]	7.3 [7.2, 7.4]	0.16
Highest serum lactic acid	5.8 [3.4, 14.5]	14.6 [10.0, 19.7]	**0.01**
Worst arterial blood gas within 24 h from ECLS initiation			
Lowest pH	7.4 [7.4, 7.5]	7.5 [7.4, 7.5]	**<0.001**
Highest serum lactic acid	1.8 [1.0, 3.4]	2.0 [1.6, 14.3]	0.076
Initial ECLS Flow (first 4 h) (mL/kg/min)	124.1 [101.9, 147.7]	152.7 [123.5, 180.1]	**0.006**
Duration of ECLS (h)	82.0 [52.5, 151.0]	138.5 (104.3, 205.3)	**0.005**
ECLS complications			
Cardiovascular	24 (77%)	32 (97%)	**0.047**
Renal	15 (48%)	21 (62%)	0.404
Hematologic	11 (36%)	18 (53%)	0.244
Mechanical	11 (36%)	6 (18%)	0.176
Neurologic	6 (20%)	8 (24%)	0.97
Pulmonary	4 (13%)	4 (12%)	1
Metabolic	1 (3%)	3 (9%)	0.651
Duration of mechanical ventilation (h)	437.6 [181.6, 683.3]	412.9 (276.7, 553.4)	0.864
Stage II or III AKI based on KDIGO score	17 (54.8%)	18 (52.9%)	1
Length of Stay (LOS) (days)			
CICU LOS	32.0 [19.0, 44.0]	17.5 (10.3, 41.0)	**0.03**
Postoperative LOS	40.0 [28.0, 95.5]	17.5 (10.3, 43.3)	**<0.001**
Hospital LOS	49.0 [37.0, 76.5]	24.5 (15.3, 49.8)	**0.001**
Operative mortality based on source of pulmonary blood flow			**0.005**
m-BTT shunt	5/22 (22.7%)	5/6 (83.3%)	
Sano shunt	7/9 (77.8%)	21/28 (75.0%))	
Overall operative mortality	12 (38.7%)	26 (76.5%)	**0.005**

Results depicted in *n* (%), median (interquartile range). Statistically significant values are indicated in bold.

Abbreviations: AA: Aortic Atresia; AS: Aortic Stenosis; DILV: Double Inlet left Ventricle; DORV: Double Outlet Right Ventricle; HLHS: Hypoplastic Left Heart Syndrome; MA/AA: Mitral Atresia/Aortic Atresia; MA/AS: Mitral Atresia/Aortic Stenosis; MS/AA: Mitral Stenosis/Aortic Atresia; MS/AS: Mitral Stenosis/Aortic Stenosis; PGE1: Prostaglandin; TTE: Transthoracic Echocardiogram; m-BTT shunt: modified Blalock Taussig Thomas shunt; LV: Left Ventricle; RV: Right Ventricle; TEE: Transesophageal Echocardiogram; VIS: Vasoactive Inotropic Score; CICU: Cardiac Intensive Care Unit.

**Table 3:** Causes of comorbidity and death, and reintervention for patients requiring ECLS post-Norwood Operation.

Cause of death	Total number of deaths *n* = 38 (%)
Multi organ failure	12 (31.6%)
Withdrawal of support/redirection of goals of care	10 (26.3%)
Cardiac arrest after ECLS decannulation	6 (15.8%)
Sepsis/septic shock	5 (13.2%)
Severe neurological injury	5 (13.2%)

Reintervention	Total number reintervention *n* = 6/65 (9.2%)

Revision of Aortic Arch/Revision of (DKS) Damus Kaye Stansel	5
Shunt revision	1

### Patient demographics and characteristics for overall cohort stratified by timing of ECLS initiation

We then stratified patients into two groups based on the timing of initiation of ECLS post-Norwood (<5 days post-Norwood vs. ≥5 days post-Norwood). Patients in the early group had higher median PaO_2_ in the first 48 h of ECLS (274.6 mmHg, IQR 166.24, 313.12 vs. 101.79 mmHg, IQR 67.70, 201.60, *p* < 0.001), had higher rates of central vs. peripheral cannulation (85% vs. 37%, *p* < 0.0001), had higher serum lactate within 2 h of ECLS initiation 12.85 (IQR 5.12, 15.23) vs. 5.74 (IQR 3.0, 12.20), *p* = 0.02, and had shorter CICU length of stay (LOS), postoperative LOS, and overall hospital LOS. A full comparison between the 2 groups is shown in Supplemental Table 1.

### Functional Status Scale (FSS)

The mean total FSS score for survivors in the non-hyperoxia group increased from 6 (SD 0) on admission/baseline to 8.4 (SD 1.2) at discharge (*p* < 0.0001), while the mean total FSS score for survivors in the hyperoxia-group increased from 6 (SD 0) on admission/baseline to 8.8 (SD 1.4) at discharge (*p* = 0.0008). Comparisons between different FSS domains on admission and discharge are presented in Table 4. The mean FSS score difference between the two groups was 0.4 (95% CI: −0.9, 1.6, *p* = 0.512). Of the 27 overall survivors, 19/27 (70%) were from the non-hyperoxia group and 8/27 (30%) were from the hyperoxia group. We failed to identify an association between designation as “hyperoxia” and new morbidity, or unfavorable outcome (Table 5).

**Table 4:** Functional Status Scale (FSS) for post-Norwood ECLS survivors on admission and discharge stratified by PaO_2_ levels into hyperoxia group (PaO_2_ > 182 mmHg) and non-hyperoxia group (PaO_2_ ≤ 182 mmHg).

ECLS group	FSS domain	Admission		Discharge		*p*-value
		Mean	SD	Mean	SD	
Non-Hyperoxia Group (PaO_2_ ≤ 182 mmHg) (*n* = 19)	Mental Status	1	0	1.1	0.5	**0.331**
	Sensory Function	1	0	1	0	**–**
	Communication	1	0	1.1	0.2	**0.331**
	Motor Functioning	1	0	1.1	0.5	**<0.0001**
	Feeding	1	0	2.9	0.5	**0.042**
	Respiratory Status	1	0	1.2	0.4	**<0.0001**
	Total Score	6	0	8.4	1.2	**<0.0001**
Hyperoxia Group (PaO_2_ > 182 mmHg) (*n* = 8)	Mental Status	1	0	1.1	0.4	0.351
	Sensory Function	1	0	1.1	0.4	0.351
	Communication	1	0	1.1	0.4	0.351
	Motor Functioning	1	0	1	0	**–**
	Feeding	1	0	3	0	**–**
	Respiratory Status	1	0	1.4	0.7	0.197
	Total Score	6	0	8.8	1.4	**0.001**

Results depicted as mean, and standard deviation (SD).

Subscale scores range from 1 to 5. Total scores are the sum of subscale scores ranging from 6 to 30.

*p*-value: paired t-tests

**Table 5:** New morbidity and unfavorable functional outcome for overall survivors who required ECLS post-Norwood operation stratified by PaO_2_ levels into hyperoxia and non-hyperoxia groups based on functional status scale change from admission to discharge.

ECLS group	New morbidity (change in FSS score ≥3 points)	Unfavorable outcome (change in FSS score ≥5 points)
Overall Cohort of Survivors (*n* = 27)	7 (26%)	2 (7%)
Non-Hyperoxia Group Survivors (*n* = 19) (PaO_2_ ≤ 182 mmHg)	4 (21%)	1 (5%)
Hyperoxia Group Survivors (*n* = 8) (PaO_2_ > 182 mmHg)	3 (38%)	1 (13%)

FSS: Functional Status Scale; ECLS: Extracorporeal Life Support.

### Outcomes analysis

**Figure 3:**
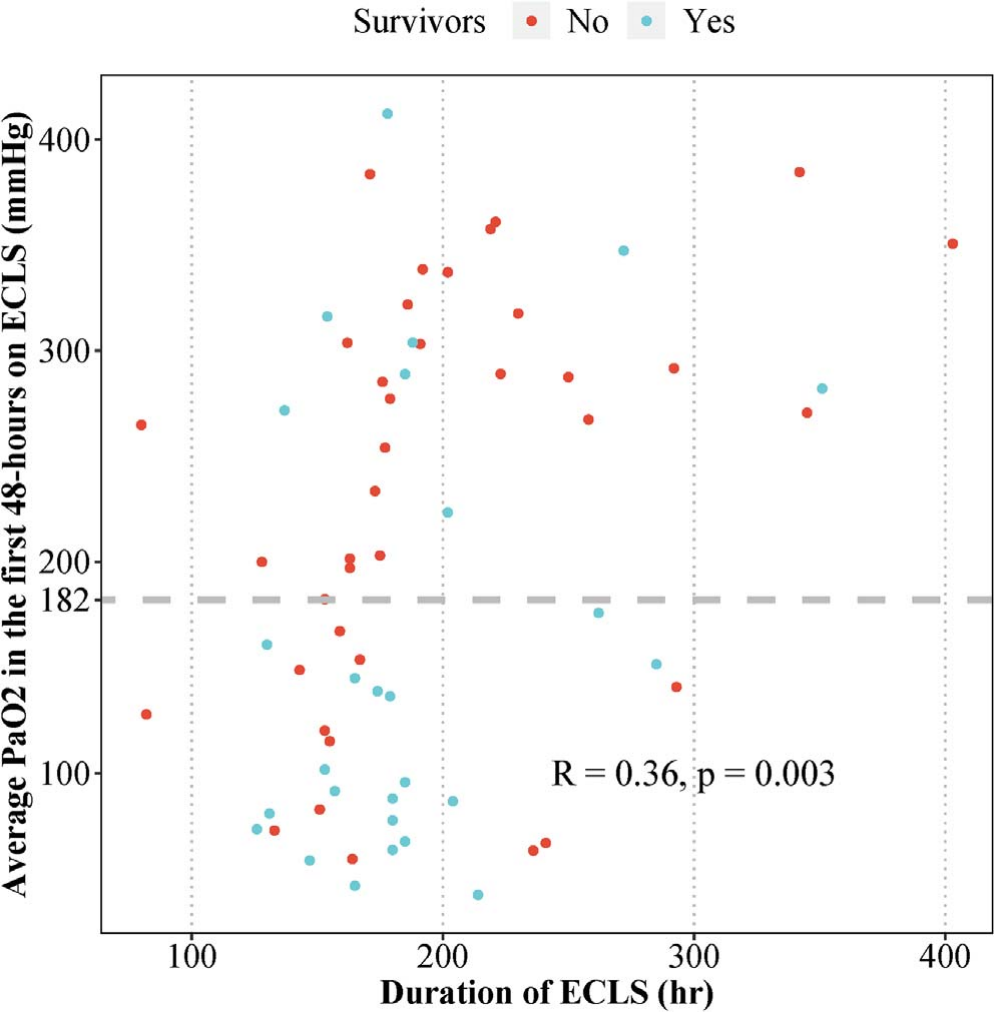
Scatterplot illustrating the association of average PaO_2_, ECLS duration, and mortality in neonates requiring ECLS support post-Norwood operation.

In univariable analysis, using the ROC curve, PaO_2_ > 182 mmHg was associated with higher odds of mortality [OR 5.2 (95% CI: 1.8–15.0), *p* = 0.003]. However, the association was insignificant when controlling for the source of pulmonary blood flow, CPB time, and post-Norwood VIS score at 48 h [OR 3.1 (95% CI: 0.8–12.3), *p* = 0.104]. No difference in stage II or III AKI or PPLOS was detected between the hyperoxia and non-hyperoxia-groups (Table 6). The association of average PaO_2_ and CPB time is graphically demonstrated in Figure 3, with a correlation coefficient of 0.4 (95% CI: 0.1–0.6, *p* = 0.003).

**Table 6:** Outcomes of neonates requiring extracorporeal life support post-Norwood operation using univariable and multivariable regression analysis.

Variables	Non-hyperoxia Group	Hyperoxia Group	OR (95% CI)	*p*-value	aOR^a^ (95% CI)	Adjusted *p*-value
	Median PaO_2_ ≤ 182 (*n* = 31)	Median PaO_2_ > 182 (*n* = 34)				
Stage II or III AKI	17 (54.8%)	18 (52.9%)	0.93 (0.35, 2.46)	0.878	1.38 (0.37, 5.13)	0.634
PPLOS^b^	12 (38.7%)	5 (14.7%)	0.27 (0.08, 0.9)	**0.033**	0.41 (0.09, 1.76)	0.229
Operative death	12 (38.7%)	26 (76.5%)	5.15 (1.76, 15.04)	**0.003**	3.11 (0.79, 12.26)	0.104

a Adjusted for source of pulmonary blood flow, CPB time, and post-Norwood VIS-score at 48 hours.

b Third quartile of postoperative length of stay (≥55) was applied as cutoff.

Abbreviations: PaO_2_: Partial Pressure of Oxygen; AKI: Acute Kidney Injury; PPLOS: Prolonged Postoperative Length of Stay.

## Discussion

Despite advances in cardiac surgery and extracorporeal technology, morbidity and mortality persist. Many of the risk factors for poor outcomes, including weight and surgical complexity are not modifiable. Thus, there is value in identifying practice-based risk factors to improve outcomes. We describe the relationship between hyperoxia in the first 48-hours while on ECLS and mortality in an unadjusted analysis with an OR of 5.15. However, this association did not persist when adjusting for confounding variables (source of pulmonary blood flow, CPB time, and post-Norwood VIS score at 48 h). It is possible that this lack of association was due to inadequate sample size. There was no significant association seen with AKI or PPLOS. Although oxygen administration was not standardized, there were important differences in the treatment groups. The hyperoxia group had different operative strategies and higher markers of illness, however received earlier ECLS with greater support.

In other critical illness settings, an association between excessive oxygen delivery with poor clinical outcomes has been reported. In patients requiring ECLS for cardiac arrest, hyperoxia (as defined by a mean PaO_2_ > 193 Torr) was associated with 30-day mortality and the need for dialysis [1, 21, 32]. Several reports of neonates with asphyxia have demonstrated an association between hyperoxia and a risk of brain injury and mortality [1, 33, 34]. In a prior report we showed that a substantial portion of infants undergoing cardiac surgery using CPB were exposed to hyperoxia and patients in the hyperoxia group had four-fold greater odds of mortality within 30 days of surgery [14]. This current report supports earlier findings that hyperoxia is likely associated with worse outcomes. However, understanding of particular populations at risk remains unclear as some studies fail to demonstrate an association between hyperoxia and mortality [35].

Despite previous studies, there is no generally accepted definition of pathologic hyperoxia. Injurious hyperoxia may vary by patient population and clinical context [32]. As we know, the oxygen content of blood consists of bound oxygen to hemoglobin, and dissolved oxygen in the form of PaO_2_. Despite knowing that the bound oxygen by far is the main contributor for oxygen content in blood, the dissolved oxygen (PaO_2_) is what is important at the cellular level. Poor outcomes may occur when PaO_2_ exceeds a certain threshold of antioxidation systems of the body. This is biologically plausible as endogenous antioxidants may prevent oxidative stress at lower PaO_2_. When high amounts of oxygen are introduced to previously ischemic tissues, this leads to the generation of reactive oxygen species (ROS) and activation of inflammatory pathways via cytokines and other immunological signaling pathways. The generation of oxygen free radicals causes damage to the cell membrane integrity due to lipid peroxidation and protein changes, ultimately resulting in premature cell death [36]. Production of ROS can result in the dysfunction of organ systems including the immune system. This dysregulation may result in multiorgan dysfunction in the form of renal failure, cardiac dysfunction, and respiratory failure. These may ultimately increase the overall risk of morbidity and mortality [37–42]. In patients who have experienced cardiac arrest or resuscitation aftershock, an increase in ROS may deplete plasma antioxidant potential which may lower the threshold for subsequent oxidative injury [32]. This effect may be more pronounced in neonates and infants as they are known to have immature antioxidant defenses and thus may be more susceptible to ROS [1]. The effect of hyperoxia may be further pronounced in patients with cyanotic heart disease as they have significantly lower PaO_2_ at baseline. It is not known if the antioxidant systems of the body are downregulated in patients with lower baseline PaO_2_. Although unknown, it is plausible that these patients are more vulnerable to supraphysiologic oxygen.

Interestingly we found that the hyperoxia cohort did not have longer times from the OR/CICU arrival to initiation of ECLS, and 4 h after initiation on ECLS, they had a higher median rate of flow (Table 2). This suggests that the hyperoxia cohort did not have a delay in support or inadequate ECLS support. However, the hyperoxia cohort conversely had a higher serum lactate prior to ECLS initiation. It is difficult to account for these differences, however they support the benefits of a prospective and controlled study.

Because there is no generally accepted definition of hyperoxia in this population, we used an ROC curve analysis to determine which PaO_2_ values may be associated with an adverse outcome. A similar strategy was employed in previous reports. Sznycer-Taub et al. evaluated hyperoxia in infant cardiac patients supported on VA-ECMO and found that a PaO_2_ of 193 Torr in the first 48 hours was determined to have an association with 30-day mortality [1]. Using a similar strategy, Beshish showed that a PaO_2_ of 313 Torr for infants undergoing cardiac surgery utilizing CPB was associated with 30-day mortality [14]. Our cut-off definition of hyperoxia was very close to that identified by Sznycer-Taub although the patient population was slightly different, as the former captured all infants on postoperative VA-ECMO. Our study supports the findings of this prior report as we evaluated a more homogenous and larger patient population who required ECLS following Norwood operation (*n* = 65) using a similar hyperoxia definition.

## Limitations

Our findings are subject to all limitations inherent to single-center retrospective cohort studies. Although PaO_2_ levels were obtained at dedicated time intervals, it is not possible to discern the effect of time spent in a hyperoxia state as opposed to the effects of acutely high PaO_2_ levels. Additionally, there may be some bias as to which patients are exposed to hyperoxia. Our center does not have a standard protocol dictating goal PaO_2_ levels, however, this study builds support for such a practice. The majority of our cohort had a PaO_2_ level over 200 mmHg while on ECLS limiting our ability to study the relationship between lower oxygen tension levels and outcomes. This data may not be generalizable to centers that target lower PaO_2_ values. The retrospective nature of this study prevented our description of other surrogate markers of cardiac output such as ECLS flows, pulsatility, or Qp:Qs. There may be concerns about generalizability, as our incidence of ECLS (24%) was higher than the 8–20% in other reports [4, 5, 7–9]. This may be due to a lower threshold for ECLS at our center, higher complexity of patients, or variations in surgical strategy. Notably, despite the higher rate of ECLS, Norwood operation survival in our center (86%) is similar to STS benchmark public outcome reporting (88%) [43, 44]. It is important to note that some baseline characteristics differed in the hyperoxia group, notably including time to cannulation and site of cannulation. It is potential that these factors were strongly associated with patient outcomes and are variables that must be considered in future investigations. It is unknown if cannulation position affects oxygen delivery and hyperoxia exposure and is a potential topic for future studies. Many of these limitations can be addressed in a multicenter validation study, which our group is currently pursuing, and ultimately a randomized controlled trial. We also acknowledge that after controlling for confounders, hyperoxia (PaO_2_ > 182 mmHg) was not associated with outcomes. This may be due to a small sample size and can be addressed in a multicenter validation study.

## Conclusions

Of the 65 patients who required ECLS post-Norwood operation, 27 (42%) survived hospital discharge. Using a derived cut point, hyperoxia was associated with 5× higher odds of mortality in unadjusted analysis. When adjusted for confounding variables, there was no association with mortality. Hyperoxia was not associated with the development of AKI, PPLOS, or a new functional status morbidity. Multicenter and prospective evaluation of this modifiable risk factor is imperative to improve the care of this high-risk cohort.

## Data Availability

All available data are incorporated into the article.

## Supplementary Material

The supplementary material of this article is available at https://ject.edpsciences.org/10.1051/ject/2024020/olm. *Supplemental Table 1*: Patient Demographics and Clinical Characteristics for Neonates Requiring Extracorporeal Life Support post-Norwood Operation Stratified by Timing of ECLS initiation (<5 days post-Norwood vs. >5 days post-Norwood).
