# Supplementary material for: Determining the association of hyperoxia while on extracorporeal life support with mortality in neonates following Norwood operation
Source: J Extra Corpor Technol. 2024 Dec 20;56(4):174–84. doi: 10.1051/ject/2024020 (PMC11661777; doi:10.1051/ject/2024020)
Supplement: Supplementary file 1 — Supplemental Table 1: Patient Demographics and Clinical Characteristics for Neonates Requiring Extracorporeal Life Support post-Norwood Operation Stratified by Timing of ECLS initiation (<5 days post-Norwood vs. >5 days post-Norwood). [file ject-56-174-s1.pdf]

Supplemental Table 1. Patient Demographics and Clinical Characteristics for Neonates Requiring Extracorporeal Life Support post-Norwood Operation Stratified by Timing of ECLS initiation (< 5 days post-Norwood vs > 5 days post-Norwood)

Variables	Early ECLS Group <5 days post-Norwood (n = 41)	Late ECLS Group >5 days post-Norwood (n = 24)	p-value
**Age** (days)	6.0 [4.0, 7.0]	5.0 (5.0, 8.0)	0.201
**Weight** (Kg)	3.25 [2.8, 3.5]	3.2 (2.7, 3.9)	0.751
**Sex**			
Male	26 (63%)	14 (58%)	0.188
Female	15 (37%)	10 (42%)	
**Race**			0.467
Caucasian	22 (54%)	9 (38%)	
African American	17 (41%)	12 (50%)	
Hispanic	2 (5%)	1 (4%)	
Other	2 (5%)	0 (3%)	
**Gestational Age** (weeks)	38.5 [37.3, 39.0]	38.0 [37.0, 39.0]	0.506
**Chromosomal Abnormality and Genetic Syndrome**	8 (20%)	9 (38%)	1
**Primary Cardiac Diagnosis**			0.897
AA & critical AS	2 (5%)	1 (4%)	
DILV	3 (7%)	1 (4%)	
DORV	0 (0%)	1 (4%)	
HLHS	32 (78%)	18 (75%)	
Single Ventricle Other	2 (5%)	1 (4%)	
Tricuspid Atresia	2 (5%)	2 (8%)	
**HLHS Variant**			0.1
MA/AA	17 (53%)	9 (50%)	
MA/AS	2 (6%)	1 (6%)	
MS/AA	10 (31%)	4 (22%)	
MS/AS	5 (16%)	3 (17%)	
**Pre-Norwood Respiratory Support**			0.759
RA	12 (29%)	6 (25%)	
NC	9 (22%)	5 (21%)	
HFNC	7 (17%)	3 (13%)	
Intubated	13 (32%)	10 (42%)	
**Pre-Norwood Transthoracic Echocardiogram**			
**Atrioventricular Valve Regurgitation (AVVR)**			0.692
Moderate – Severe	7 (17%)	4 (17%)	
No – Trivial – Mild AVVR	34 (83%)	20 (83%)	
**Systemic Ventricular Function**			0.055
Normal	17 (41%)	16 (67%)	
Mild Dysfunction	7 (17%)	5 (21%)	
Moderate – Severe Dysfunction	16 (39%)	3 (13%)	
**Pre-Norwood Ascending Aorta Diameter (mm)**			
Diameter (mm)	2.55 [1.93, 4.65]	2.50 [2.10, 4.80]	0.410
Median z-score	-4.00 [-4.52, -2.64]	-3.92 [-4.36, -2.54]	0.367
**Pre-Norwood Ascending Aorta Groups Based on Diameter**			0.868
≤ 1.5 mm	3 (7%)	0 (0%)	
1.6 – 1.9 mm	10 (24%)	6 (25%)	
2.0 – 3.9 mm	16 (39%)	9 (38%)	
≥ 4.0 mm	12 (29%)	9 (38%)	
**Source of Pulmonary Blood Flow**			0.133
m-BTT shunt	15 (37%)	14 (58%)	
Sano Shunt	26 (63%)	10 (42%)	
**Type of Systemic Ventricle**			0.833
LV	8 (20%)	6 (25%)	
RV	32 (78%)	18 (75%)	
undetermined	1 (2%)	0 (0%)	
**Intraoperative Transesophageal Echocardiogram**			
**Atrioventricular Valve Regurgitation (AVVR)**			0.323
Moderate – Severe	11 (27%)	6 (71%)	

Supplemental Table 1. Patient Demographics and Clinical Characteristics for Neonates Requiring Extracorporeal Life Support post-Norwood Operation Stratified by Timing of ECLS initiation (< 5 days post-Norwood vs ≥ 5 days post-Norwood)

Variables	Early ECLS Group <5 days post-Norwood (n = 41)	Late ECLS Group ≥5 days post-Norwood (n = 24)	p-value
No – Trivial – Mild AVVR	30 (73%)	18 (29%)	
**Systemic Ventricular Function**			0.390
Normal	22 (54%)	15 (63%)	
Mild Dysfunction	10 (24%)	6 (24%)	
Moderate – Severe Dysfunction	9 (22%)	3 (12%)	
**Cardiopulmonary Bypass Time** (min)	182.5 [168.0, 222.5]	177.0 [153.0, 214.0]	0.334
**Cross Clamp Time** (min)	70.0 [55.0, 85.0]	77.0 [62.0, 94.0]	0.538
**Circulatory Arrest Time** (min)	3.5 [2.0, 15.5]	8.0 [2.0, 24.0]	0.448
**Post-Norwood iNO on Arrival to CICU**	11 (27%)	5 (21%)	0.671
**Post-Norwood VIS-Score**			
First 24 hours	25.0 [20.0, 30.0]	20.0 (15.0, 27.0)	0.107
Hours 24 – 48	30.0 [20.25, 38.0]	23.0 (18.0, 28.0)	0.143
**Delayed Sternal Closure**	33 (80%)	17 (71%)	0.708
**Median PaO_2 in first 48 hours of ECLS** (mmHg)	274.61 [166.24, 313.12]	101.79 [67.70, 201.60]	**<0.0001**
**Cannulation Site**			**<0.001**
Peripheral	6 (15%)	15 (63%)	
Central	35 (85%)	9 (37%)	
**ECLS Indication**			0.832
E-CPR	17 (41%)	10 (42%)	
Cardiac	24 (59%)	14 (58%)	
**Arterial Blood Gas within 2 hours from ECLS Initiation**			
Lowest pH	7.32 [7.25, 7.39]	7.21 [7.15, 7.28]	**0.002**
Highest Serum Lactic Acid	12.85 [5.12, 15.23]	5.74 [3.0, 12.20]	**0.002**
**Worst Arterial Blood Gas within 24 hours from ECLS Initiation**			
Lowest pH	7.45 [7.41, 7.50]	7.44 [7.39, 7.46]	0.201
Highest Serum Lactic Acid	2.05 [1.47, 3.45]	1.80 [1.16, 3.40]	0.102
**Initial ECLS Flow (1st 4 hours) (ml/kg/min)**	144.6 [125.0, 157.1]	125.0 [129.6, 135.9]	0.241
**Duration of ECLS** (hours)	134.5 [89.5, 177.8]	99.0 (58.0, 169.0)	0.214
**ECLS Complications**			
Cardiovascular	35 (85%)	16 (67%)	0.086
Renal	22 (54%)	11 (46%)	0.683
Hematologic	18 (44%)	8 (33%)	0.739
Mechanical	6 (15%)	10 (42%)	**0.008**
Neurologic	10 (24%)	4 (17%)	0.751
Pulmonary	4 (10%)	3 (13%)	0.696
Metabolic	2 (5%)	1 (4%)	0.1
**Duration of Mechanical Ventilation** (hours)	401.1 [252.8, 563.2]	531.4 (308.6, 761.9)	0.089
**Stage II or III AKI based on KDIGO score**	24 (59%)	8 (33%)	0.064
**Length of Stay (LOS)** (days)			
CICU LOS	19.5 [14.8, 38.5]	46.0 [37.8, 63.3]	**0.003**
Postoperative LOS	19.0 [11.3, 47.0]	36.0 [23.0, 47.0]	**0.036**
Hospital LOS	19.5 [14.75, 42.5]	46.0 [42.3, 70.5]	**0.004**
**Operative Mortality Based on Source of Pulmonary Blood Flow**			0.398
**m-BTT Shunt**	5/15 (33.3%)	5/14 (35.7%)	
**Sano Shunt**	18/26 (69.2%)	7/10 (70.0%))	
**Overall Operative Mortality**	23 (56.1%)	12 (50.0%)	0.800

Results depicted in n (percent), median (interquartile range)

Abbreviations: AA: Aortic Atresia; AS: Aortic Stenosis; DILV: Double Inlet left Ventricle; DORV: Double Outlet Right Ventricle; HLHS: Hypoplastic Left Heart Syndrome; MA/AA: Mitral Atresia/Aortic Atresia; MA/AS: Mitral Atresia/Aortic Stenosis; MS/AA: Mitral Stenosis/Aortic Atresia; MS/AS: Mitral Stenosis/Aortic Stenosis; PGE1: Prostaglandin; TTE: Transthoracic Echocardiogram; m-BTT shunt: modified Blalock Taussig Thomas shunt; LV: Left Ventricle; RV: Right Ventricle; TEE: Transesophageal Echocardiogram; VIS: Vasoactive Inotropic Score; CICU: Cardiac Intensive Care Unit.
